# Revealing the structure of the active sites for the electrocatalytic CO_2_ reduction to CO over Co single atom catalysts using *operando* XANES and machine learning

**DOI:** 10.1107/S1600577524004739

**Published:** 2024-06-25

**Authors:** Andrea Martini, Janis Timoshenko, Martina Rüscher, Dorottya Hursán, Mariana C. O. Monteiro, Eric Liberra, Beatriz Roldan Cuenya

**Affiliations:** a Department of Interface Science Fritz Haber Institute of the Max Planck Society 14195Berlin Germany; ESRF – The European Synchrotron, France

**Keywords:** CO2RR, single-atom catalysts, *operando* XAS, machine learning, data analysis

## Abstract

Transition-metal nitro­gen-doped carbons (TM-N-C) are emerging as a highly promising catalyst class for several important electrocatalytic processes, including the electrocatalytic CO_2_ reduction reaction (CO_2_RR). The unique local environment around the singly dispersed metal site in TM-N-C catalysts is likely to be responsible for their catalytic properties, which differ significantly from those of bulk or nanostructured catalysts. However, the identification of the actual working structure of the main active units in TM-N-C remains a challenging task due to the fluctional, dynamic nature of these catalysts, and scarcity of experimental techniques that could probe the structure of these materials under realistic working conditions. This issue is addressed in this work and the local atomistic and electronic structure of the metal site in a Co–N–C catalyst for CO_2_RR is investigated by employing time-resolved *operando*X-ray absorption spectroscopy (XAS) combined with advanced data analysis techniques. This multi-step approach, based on principal component analysis, spectral decomposition and supervised machine learning methods, allows the contributions of several co-existing species in the working Co–N–C catalysts to be decoupled, and their XAS spectra deciphered, paving the way for understanding the CO_2_RR mechanisms in the Co–N–C catalysts, and further optimization of this class of electrocatalytic systems.

## Abstract 2

*Operando* XANES analysis assisted by machine learning, spectral decomposition approaches and DFT modelling is employed to shed light on the speciation of Co and N co-doped carbon catalyst during electrocatalytic CO_2_ conversion.

## 1. Introduction

Transition-metal nitro­gen-doped carbon (TM-N-C) catalysts are currently attracting great interest because of their promising performance in several electrocatalytic processes, such as oxygen reduction reactions (Zitolo *et al.*, 2015 ▸, 2017 ▸), nitrate reduction (Wu *et al.*, 2021 ▸; Wang *et al.*, 2022 ▸) and electrocatalytic CO_2_ reduction (CO_2_RR) (Liang *et al.*, 2021 ▸; Genovese *et al.*, 2018 ▸; Li *et al.*, 2022 ▸). The interest is based not only on the excellent catalytic properties of TM-N-C materials but also on their potential to decrease our dependence on precious metal resources and optimize the utilization of metal atoms (Hursán *et al.*, 2024 ▸). It is commonly believed that in the as-prepared TM-N-C catalysts the metal species are anchored to the carbon matrix by four N atoms, although some anchoring structures with lower coordination are reported in the literature as well (Fan *et al.*, 2020 ▸; Yang *et al.*, 2018 ▸; Wang *et al.*, 2018 ▸; Gong *et al.*, 2020 ▸). In the context of CO_2_RR, Ni–N–C is the most popular choice for the CO_2_ reduction to CO, showing high selectivity [Faradaic efficiency FE_CO_ > 90% (Hursán *et al.*, 2024 ▸)] and a large activity (current density) for this process (Liang *et al.*, 2021 ▸). Interestingly, while there is consensus in the literature regarding the origin of the catalytic performance of the Ni–N–C system, the same does not apply to the case of the Co–N–C catalyst, where the observed selectivity has been shown to vary strongly among different studies, with FE_CO_ values between 20% and 100% (Ju *et al.*, 2017 ▸; Pan, Deng *et al.*, 2018 ▸; Wang *et al.*, 2018 ▸; Pan, Lin *et al.*, 2018 ▸). On one hand, this suggests that the Co–N–C catalysts can be potentially optimized to reach the same high performance for CO_2_RR as Ni–N–C, or even outperform the latter. On the other hand, the large discrepancy between the results obtained in different studies suggests that the dynamic processes taking place in the working Co–N–C catalysts can be more complex than those in the Ni–N–C system, and need to be understood in detail before the Co–N–C can be used as a practical catalyst.

*Operando*X-ray absorption spectroscopy (XAS) measurements are a suitable tool for this purpose, providing valuable insights into the oxidation state and the local electronic and atomistic structure of the Co sites under applied potential. In particular, the analysis of the extended X-ray absorption fine-structure (EXAFS) region of the XAS spectrum has been proven to be an invaluable tool for understanding the structure of working electrocatalysts. (Timoshenko & Roldan Cuenya, 2021 ▸). However, for such disordered structures as TM-N-C catalysts, the information content in the EXAFS data is limited. Moreover, the EXAFS data quality of TM-N-C catalysts is often compromised by the low metal loading, by the signal attenuation due to the electrolyte and by the formation of gas bubbles.

An alternative approach consists of focusing instead on the X-ray absorption near-edge structure (XANES) part of the XAS spectrum. XANES usually has a higher signal-to-noise ratio and is highly sensitive to the electronic structure of the absorber, but also to the details of the local 3D geometry. However, the quantitative analysis of XANES features is challenging (Guda *et al.*, 2021 ▸). In contrast to the EXAFS case, there is no analytical equation describing the XANES part (Guda *et al.*, 2019 ▸). Nonetheless, the development of *ab initio* codes for XANES simulations and of machine learning methods allows this issue to be addressed. For instance, Zitolo *et al.* (2015 ▸, 2017 ▸) utilized the *MXAN* code (Benfatto *et al.*, 2021 ▸) to fit the XANES spectra of Co–N–C and Fe–N–C compounds used for the oxygen reduction reaction. Similarly, Xiang *et al.* (2022 ▸) employed a neural network approach to decipher the XANES spectrum of a Co–N–C catalyst for the photoreduction reaction of CO_2_. Crucially, these approaches work only for phase-pure catalysts, and are not applicable for tracking the evolution of working catalysts, where the co­existence of different species or states of the metal site is expected. In our recent work on the characterization of Ni–N–C catalysts (Martini *et al.*, 2023 ▸) we addressed this problem through a multi-step approach. We first identified the number of different coexisting metal species, their corresponding kinetic profiles and XANES spectra using unsupervised machine learning methodologies (Martini & Borfecchia, 2020 ▸; Timoshenko & Frenkel, 2019 ▸). Afterwards, we deduced the atomistic structures for each of the identified species through a XANES fitting procedure facilitated by a supervised machine learning approach (Martini, Guda *et al.*, 2020 ▸; Guda *et al.*, 2019 ▸). In this study, we will extend the methodology developed for the Ni–N–C catalyst to explore the structures and behaviour of Co–N–C catalysts. Specifically, we aim to elucidate the evolution of cobalt centres during the CO_2_RR and understand their electronic and structural characteristics. By combining principal component analysis with spectral decomposition techniques we identified the distinct contributions from coexisting cobalt species to the averaged XANES spectra. The obtained spectra for pure species are interpreted using XANES simulations, including those carried out within the time-dependent density functional theory (DFT) framework, XANES data fitting and EXAFS analysis. Taken together, these techniques ultimately confirm the interactions between singly dispersed Co sites and the CO adsorbates, demonstrating the key role of these species for the electrocatalytic conversion of CO_2_ to CO. Nonetheless, our analysis suggests that these singly dispersed Co sites coexist in the working catalysts with partially reduced agglomerates of Co atoms, which limit the efficiency of Co–N–C catalysts for CO_2_RR.

## 2. Experimental and methods

### 2.1. Sample preparation

The Co–N–C catalysts were synthesized using an impregnation-calcination method (Hursán *et al.*, 2024 ▸) starting from a zeolitic imidazolate framework (ZIF-8) precursor. ZIF-8 crystals underwent carbonization in an Ar atmosphere at 1000°C for 1 h. At this temperature the majority of metallic Zn evaporated, resulting in a porous (Zn)-N-doped carbon structure, referred to as N–C. To eliminate all crystalline Zn species from the N–C support, a room temperature acid wash was performed with 20 wt% nitric acid (HNO_3_, ≥65%, Carl Roth) over 24 h. The treated sample underwent thorough washing and vacuum filtration with ultrapure water (at least 3 × 600 ml) until the pH of the supernatant solution exceeded 5.

For the synthesis of Co–N–C, 200 mg of N–C was dispersed in a 20 ml solution containing 6 m*M* of Co-nitrate [Co(NO_3_)_2_·6H_2_O, Sigma-Aldrich, ≥98%). The suspension underwent sonication for 2 h in an ultrasonic bath at ∼30–40°C and stirring for an additional 2 h at room temperature. The Co-impregnated N–C was collected through centrifugation, dried at 60°C in air, and subsequently heat-treated in Ar atmosphere at 700°C for 1 h. The final Co–N–C catalysts were obtained after an additional washing with ultrapure water and drying.

The corresponding potential-dependent CO_2_RR selectivity trends retrieved for this sample are reported in our previous work (Hursán *et al.*, 2024 ▸).

### 2.2. Experimental setup and X-ray absorption measurements

*Operando* Co *K*-edge XAS measurements were performed in our home-built single-compartment electrochemical cell (Timoshenko & Roldan Cuenya, 2021 ▸; Martini *et al.*, 2023 ▸) in fluorescence mode. The Co–N–C sample was spray-coated over a carbon paper acting as both a working electrode and a window for the X-ray fluorescence and the incoming X-rays. A Pt mesh and a leak-free Ag/AgCl electrode were used as a counter electrode and as a potential reference, respectively. The continuous flow of the electrolyte – CO_2_-saturated 0.1 *M* KHCO_3_ – was ensured by a peristaltic pump. The applied potential was controlled by a BioLogic potentiostat and was set to −1.2 V_RHE_. XAS measurements were carried out at the SAMBA beamline of the SOLEIL synchrotron radiation facility (Saint-Aubin, France). A Si(220) monochromator was used for the energy selection, while the XAS data were collected in fluorescence mode using an energy-selective 32-channel Ge detector. Alignment, background subtraction and normalization of the collected XANES data were performed using the *Athena* software (Ravel & Newville, 2005 ▸).

### 2.3. Quantitative XANES analysis

#### 2.3.1. PCA and spectral decomposition of XANES dataset

The principal component analysis (PCA) and the spectral decomposition of the XANES dataset were performed using the *PyFitIt* code (Martini, Guda *et al.*, 2020 ▸). The identification of the number of pure species contributing to the dataset was performed by examining the extracted principal components (PCs) and keeping only those *k* PCs with distinct spectral features. Additionally, four quantitative tests [scree plot, Malinowsky indicator factor (IND), imbedded error (IE) and Fisher test (Malinowski, 2002 ▸; Manceau *et al.*, 2014 ▸)] were used to further validate the number of pure species. The details of these statistical tests are discussed by Martini *et al.* (2023 ▸).

To convert the obtained PCs into the spectra of pure species we relied on a transformation matrix (TM) approach (Smolentsev *et al.*, 2009 ▸; Martini, Guda *et al.*, 2020 ▸; Martini *et al.*, 2021 ▸; Martini & Borfecchia, 2020 ▸). Here the dataset containing the original XANES spectra *X* was decomposed as *X* = 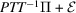. Matrix *P* contains the *k* significant PCs identified as discussed above, while the rows of Π, referred to here as PC weights, are the projections of *X* onto each of the PCs. *T* is a *k* × *k* matrix whose elements can be varied until a set of chemical/physical interpretable spectra *S* = *PT* and concentration profiles *C* = *T*^−1^Π, satisfying the imposed constraints (*i.e.*, non-negativity of the XANES and concentration profiles and mass balance condition), are identified. Finally, the error matrix 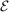 contains the residuals between the original XANES spectra in *X* and their approximations using *k* PCs.

#### 2.3.2. XANES fitting procedure

Identification of 3D structural motifs corresponding to the XANES spectra for the pure species was carried out based on the EXAFS analysis and XANES data fitting. The latter was facilitated by the indirect supervised machine learning (ML) approach, as implemented in the *PyFitIt* code (Martini, Guda *et al.*, 2020 ▸). Herein, the *FMDNES* code (Joly, 2001 ▸; Guda *et al.*, 2015 ▸) is used to calculate explicitly the XANES spectra for a few hundreds (or in some cases thousands) of relevant structure models, with different values for those structure parameters that characterize the environment around the single metal site. These structures for the ML training are chosen based on an adaptive sampling approach (active learning) (Tereshchenko *et al.*, 2022 ▸). Note that the *FDMNES* code has been demonstrated to be very accurate in the reproduction of the Co *K*-edge (Shapovalova *et al.*, 2021 ▸). *FDMNES* simulations were performed by employing the finite difference method. Real Hedin–Lundqvist and von Barth local exchange correlation potentials (Hedin & Lundqvist, 1970 ▸; von Barth & Hedin, 1972 ▸) were used. The cluster size was set to 5.5 Å. The increase of cluster size did not result in significant changes in the simulated spectra. All theoretical XANES data were aligned by correcting each energy grid by their related EPSII parameter (Joly, 2021 ▸). At the same time, a common shift of 142 eV was applied to all of them as described by Martini *et al.* (2023 ▸).

The obtained theoretical spectra are then used to train an ML model, establishing a link between the values of the structure parameters and spectral features. The trained ML is then able to generate the corresponding XANES spectra for the intermediate values of the structure parameters that were not explicitly included in the training dataset, thus allowing us to obtain theoretical XANES much faster than in the *FDMNES* simulations, providing a significant speed-up for XANES data fitting. Our ML model, describing a XANES spectrum 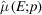 as a function of the energy and of the structural parameters *p*, consists of a set of radial basis functions (RBF),
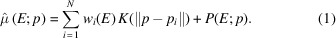
Here the 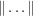 operator is the *L*_2_ norm, *K*(*r*) is a linear radial basis function while *P*(*E*;*p*) is a second-order polynomial with energy-dependent coefficients (Guda *et al.*, 2021 ▸). The unknown factors *w*_*i*_ and the polynomial coefficients are obtained during the ML training using the ridge regression. The accuracy of the trained ML model is tested using a ten-fold cross validation technique. See Martini *et al.* (2023 ▸), Martini, Guda *et al.* (2020 ▸) and Guda *et al.* (2021 ▸) for additional details on the ML training process.

The fit of the XANES spectra derived from the experimental measurements was performed using the coordinate descendent algorithm (Wright, 2015 ▸), minimizing the *R*_factor_ between the experimental and the ML-derived XANES spectra, defined as
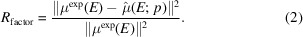
The uncertainties were estimated by calculating the intervals of the structure parameter values which would result in an increase of the residual by less than 10%.

#### 2.3.3. Pre-edge simulation

For the Co *K*-edge XANES pre-edge simulations, time-dependent DFT (TDDFT) calculations, implemented in the *ORCA* 5.0.4 code (Neese, 2012 ▸, 2022 ▸; Neese *et al.*, 2020 ▸), were employed. We used the CAM-B3LYP functional (Yanai *et al.*, 2004 ▸) with the D3BJ dispersion correction (Grimme *et al.*, 2011 ▸) using the ZORA-def2-TZVP (vanLenthe *et al.*, 1996 ▸; Schäfer *et al.*, 1992 ▸; Weigend & Ahlrichs, 2005 ▸) with the SARC/J auxiliary basis set (Rolfes *et al.*, 2020 ▸). The calculated XANES pre-edge transitions for a Co +2 state (doublet and quadruplet) were shifted by 17.1 eV until they matched the experimental pre-edge features and then broadened with 2 eV FWHM Lorentzian functions.

## 3. Results and discussion

**Figure 1:**
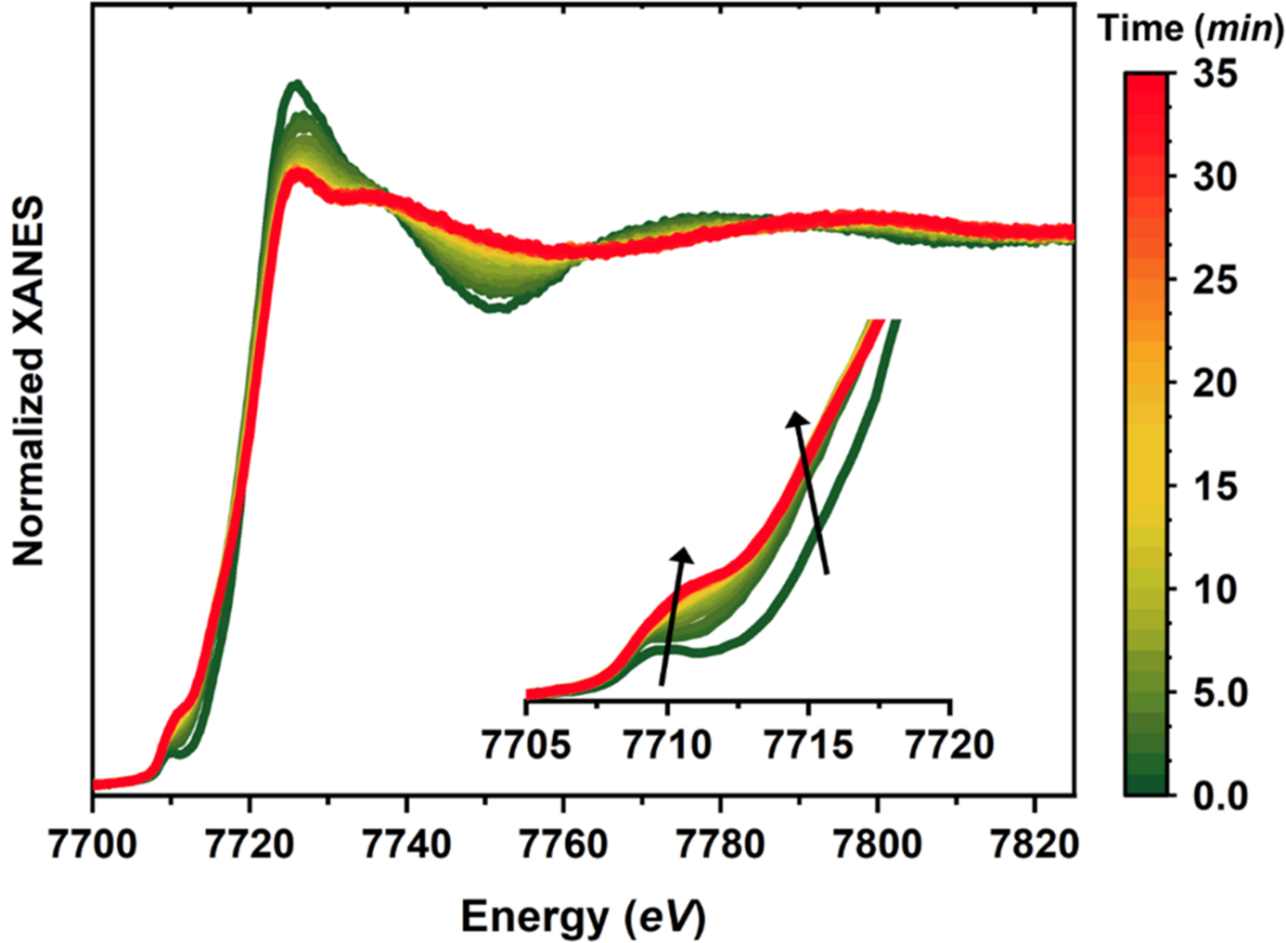
Co *K*-edge XANES dataset (27 spectra) collected under *operando* conditions during the CO_2_RR process. The inset shows a magnification of the pre-edge region. The arrows highlight the evolution of the XANES features with time under reaction conditions. Measurements performed at −1.2 V_RHE_ in 0.1 *M* KHCO_3_ as electrolyte.

Fig. 1 ▸ shows the Co *K*-edge XANES data for the as-prepared catalyst, and their evolution during the CO_2_RR. The obtained spectra for the as-prepared state match well with those in our prior work (Hursán *et al.*, 2024 ▸), but the new dataset allows us to track the changes in the catalyst structure as a function of the reaction time, thus providing valuable information about the possible reaction intermediates.

As discussed in our previous study (Hursán *et al.*, 2024 ▸), the spectrum collected at open circuit potential conditions before the reaction started, labelled as *t* = 0 min, can be associated with octahedrally coordinated Co sites, with the local geometry resembling that of an Ni–N–C catalyst (Martini *et al.*, 2023 ▸). As the reaction proceeds, the XANES white-line intensity decreases, while the pre-edge increases (see the inset of Fig. 1 ▸). Changes in the post-edge region of the XANES spectra indicate strong transformations in the catalyst atomistic structure. The observed changes can be associated with a reduction of the Co coordination number and/or the increased distortion of the local geometry.

**Figure 2:**
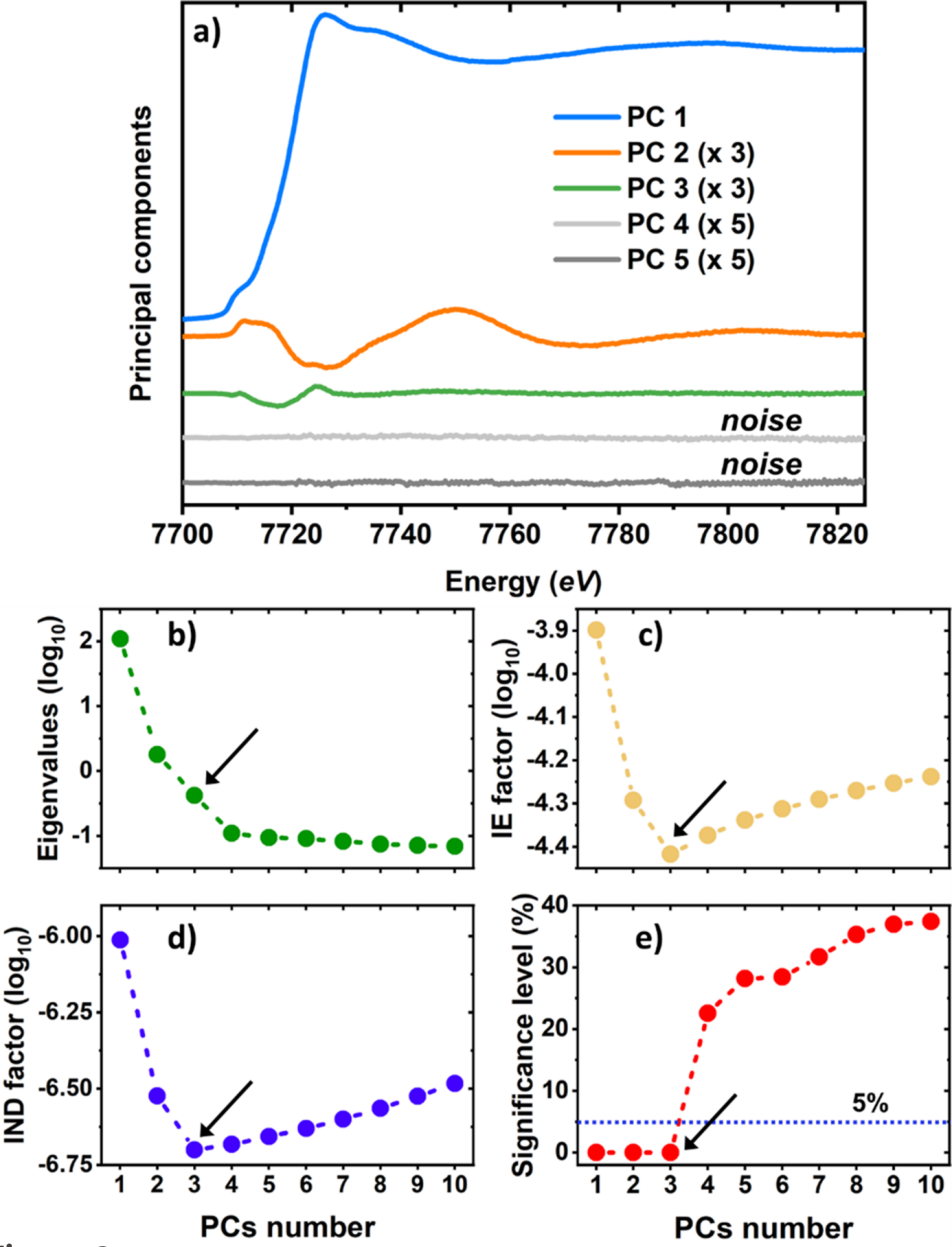
(*a*) First five principal components (PCs) extracted from the Co *K*-edge XANES dataset, weighted by their corresponding singular values. The numbers in brackets indicate the multiplicative factors used to enhance the PCs features for a clearer visualization. (*b*) Scree plot. (*c*) Imbedded error plot. (*d*) Malinowski indicator factor plot. (*e*) Significance level plot derived from the Fisher test. The arrows point to the number of pure species contributing to the XANES dataset, as obtained from each test. For all of them, this number was found to correspond to 3.

To understand the number of different species contributing to the collected dataset, and to reveal their nature, we relied first on the PCA approach. The visual analysis of each PC, see Fig. 2 ▸(*a*), indicates that only the first three PCs exhibit meaningful spectral features. This suggests that only three spectroscopically distinct species are contributing to the collected XANES dataset. This conclusion is confirmed also by the analysis of the scree plot, IND and IE indicators, and also by the Fisher test, see Figs. 2(*b*)–2(*e*).

**Figure 3:**
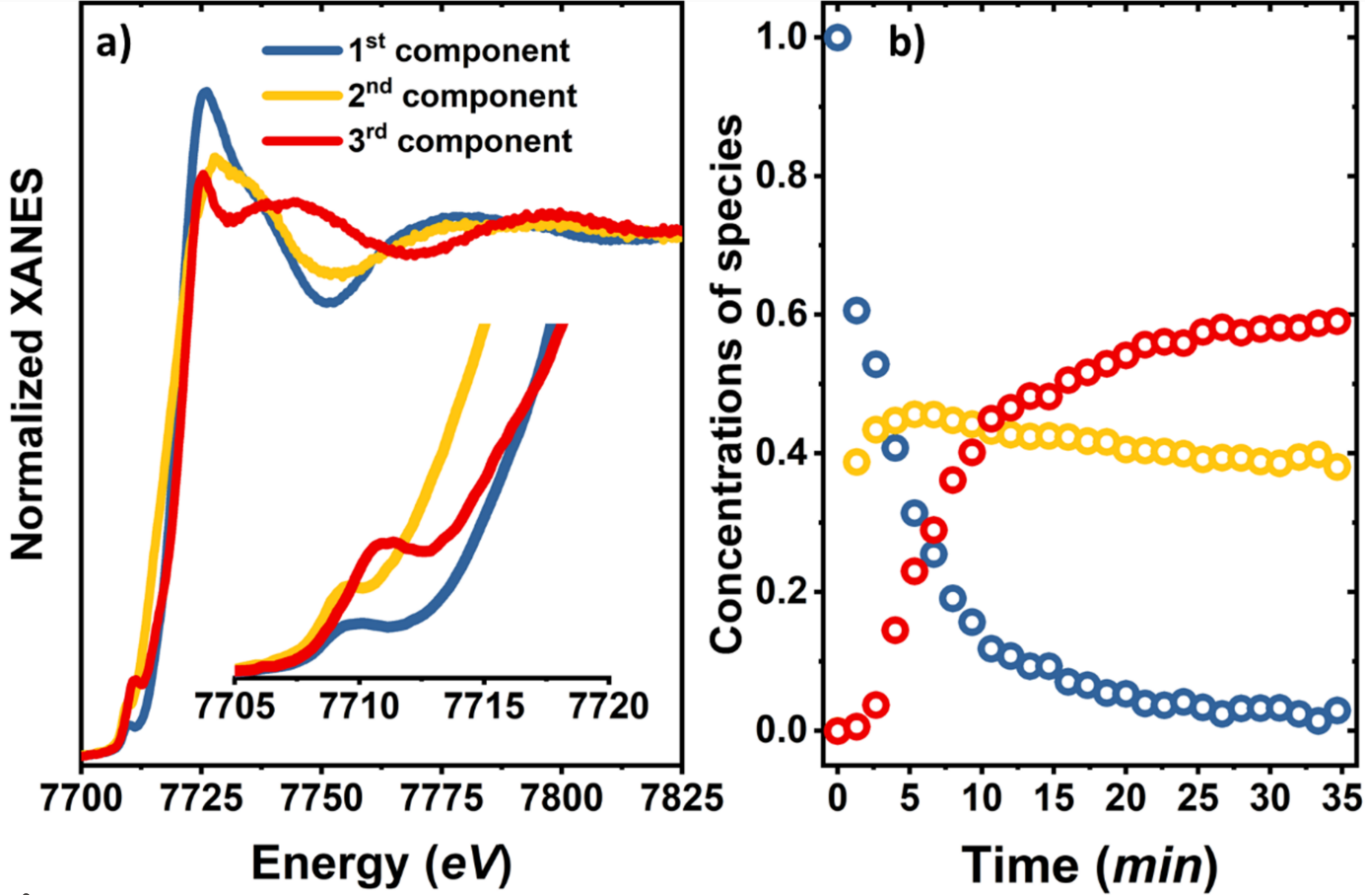
Pure XANES spectra (*a*) and concentration profiles (*b*) extracted by the TM approach. The inset in (*a*) shows a magnification of the pre-edge region for the three XANES components.

We note here that the PCs themselves do not correspond to the spectra of the pure species. Nonetheless, the latter can be obtained as some linear combination of the PCs. The spectra for the pure species and the corresponding concentration profiles can be retrieved by using the TM approach. Considering that the PCA revealed the presence of three spectroscopically distinct species, the TM matrix contains 3 × 3 = 9 unknown coefficients (projections of each of the pure species on each of the significant PCs). This number of unknowns can be further reduced by applying a set of constraints. First, we note that for the normalized XANES spectra, the weight of the first PC should be the same for all spectra in the dataset, and should be equal also to the weight of this PC to the spectra of the pure species (Martini, Guda *et al.*, 2020 ▸). The latter enforces also the mass balance condition, *i.e.*, that for each experimental XANES in the dataset the sum of the concentrations of all pure species has to be equal to 1 (Martini & Borfecchia, 2020 ▸). This useful property is satisfied if the normalization of the experimental XANES spectra 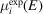 is carried out so that the quantity
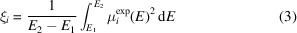
is the same for all spectra. Here *E*_1_ and *E*_2_ define the limits of the energy range used for the analysis (7700 eV and 7825 eV in our case). Setting the first row of *T* to be equal to ξ_*i*_ thus ensures that both the normalization of the XANES spectra and mass balance condition are satisfied, and reduces the number of unknown elements in *T* from 9 to 6. Finally, a further constraint was imposed: we assumed that the first spectrum of our dataset (*t* = 0 min) corresponds to one of the pure species. This assumption is plausible since this spectrum corresponds to a stable state of the as-prepared sample. The projections of the first spectrum in our dataset on the PCs thus provides us with the values of the coefficients in the first column of *T*, and reduces the number of unknown coefficients to 4. The ranges of possible values for these unknown coefficients can be further reduced to ensure that the spectra for the pure species and the concentration profiles are non-negative. The obtained spectra for the pure species and the corresponding concentration profiles are shown in Fig. 3 ▸, and their validation will be provided in the following paragraphs.

The obtained XANES spectra for the first and third pure species in the working Co–N–C catalyst resemble strongly the pure spectra isolated using the same technique for the Ni–N–C under similar reaction conditions (Martini *et al.*, 2023 ▸). Based on our prior works (Martini *et al.*, 2023 ▸; Hursán *et al.*, 2024 ▸) we thus conclude that the first species (as-prepared state of the catalyst) can be associated with a Co site surrounded by four N atoms within the carbon ring plane and two axial O atoms or OH groups. We validate this assumption using Co *K*-edge XANES data fitting, which is discussed in detail in Section S6.1 of the supporting information. The third XANES component can be tentatively attributed, instead, to a more distorted environment for single cationic Co sites where the axial O atoms of the first component have been substituted by two CO. By analogy with our observations for the Ni–N–C system, we associate this species with the main active site for CO_2_RR. We confirm and investigate its structure in more detail below. However, we first need to comment on the second pure species, identified by the TM approach, whose XANES spectrum does not resemble the spectra of the pure species obtained during CO_2_RR for the Ni–N–C catalyst (Martini *et al.*, 2023 ▸).

The XANES spectrum for this species exhibits the first pre-edge peak at ∼7709 eV, *i.e.*, at lower energies than for the first and third component, which could suggest a more reduced chemical state of Co. Moreover, the second XANES feature at ∼7716 eV is more intense compared with that in the XANES spectra for the other pure species, see the inset of Fig. 3 ▸(*a*) and Fig. S2 of the supporting information. The intense second pre-edge peak could be associated with the Co 1*s* → 4*p*_*z*_ shakedown transition and can suggest the presence of a four- or three-coordinated distorted geometry (Colpas *et al.*, 1991 ▸; Baker *et al.*, 2017 ▸). Further clues about the nature of this site can be extracted from the EXAFS analysis.

Since the concentration profiles for all species are known from the XANES analysis, the EXAFS spectra can be extracted from the original dataset as *S*_XAS_ = *X*_XAS_*C*(*CC*^*t*^)^−1^, where *t* denotes the matrix transpose and *X*_XAS_ contains absorption spectra extended to 8290 eV and normalized in the same way as used for XANES data processing. The obtained EXAFS spectra for all three species are shown in Fig. S3.

**Figure 4:**
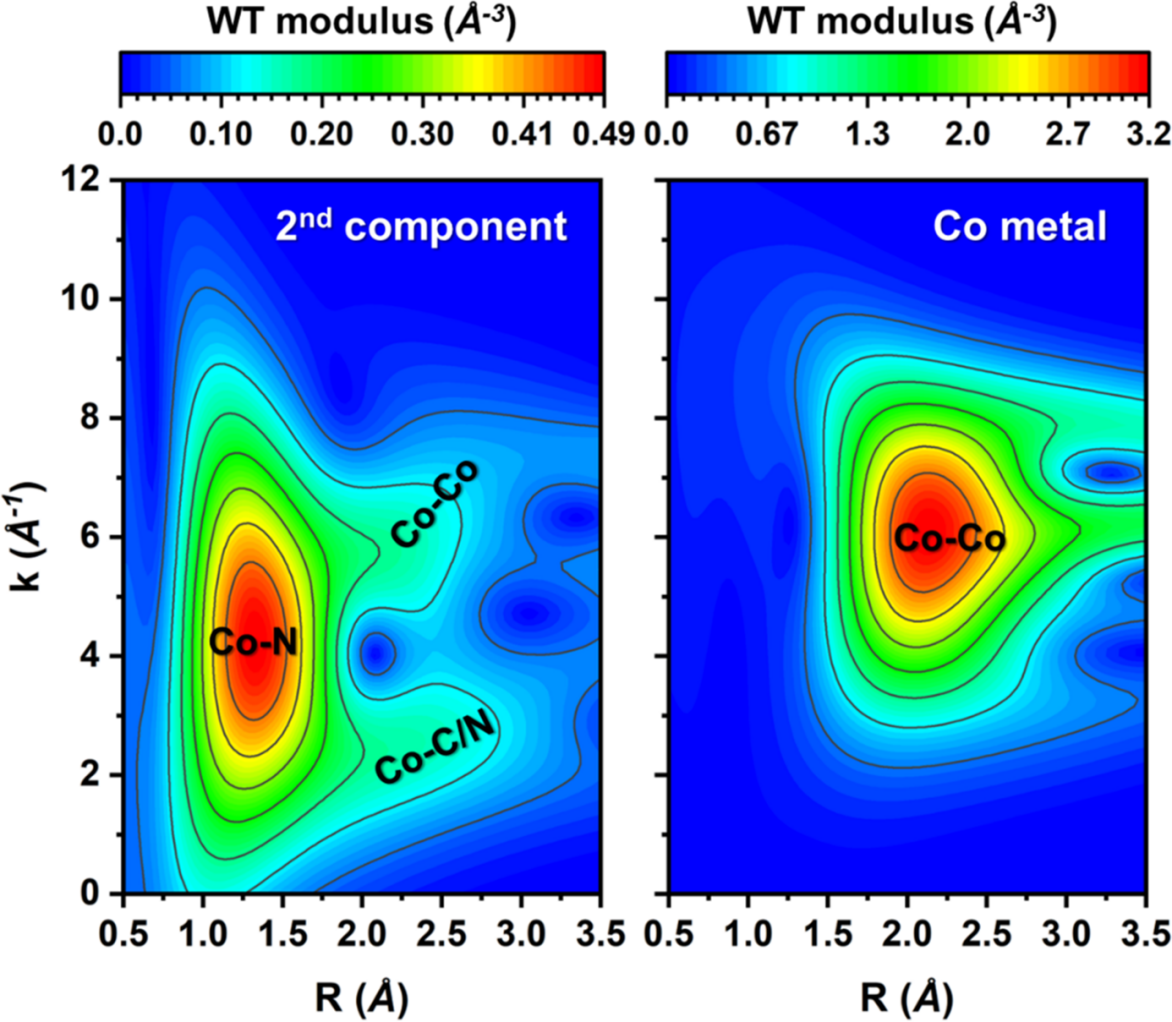
Moduli of the Morlet wavelet transform calculated for the EXAFS signal of the second pure species (*a*) and for the metallic Co foil (*b*). The wavelet resolution parameters σ and η were set to 1 and 6, respectively. The wavelet transforms are not corrected for the phase shift.

For the qualitative interpretation of the EXAFS data we relied on wavelet transform (WT) analysis (Timoshenko & Kuzmin, 2009 ▸; Funke *et al.*, 2005 ▸; Martini, Signorile *et al.*, 2020 ▸) (see Section S5.1 for more technical details). While for the first and third pure species the WT-EXAFS spectra are consistent with those expected for the singly dispersed cationic site (Martini *et al.*, 2023 ▸; Hursán *et al.*, 2024 ▸), for the second component [Fig. 4 ▸(*a*)] we observe an intense contribution of the second coordination shell in the *R* range between 2 and 2.7 Å, which appears to be split into two regions in the *k*-space, namely 1–3 Å^−1^ and 6–8 Å^−1^. The contribution at lower *k* values can be attributed to Co—C (or Co—O) and Co—N bonds. On the other hand, the position of the second contribution both in *R*- and *k*-spaces resembles that of Co—Co bonds in metallic Co, see Fig. 4 ▸(*b*), suggesting the existence of Co—Co moieties.

In order to confirm this conclusion, we performed EXAFS data fitting. Details on the EXAFS fitting and the complete list of the refined parameters can be found in Section S5.2 and in Table S2. The fit indicates the existence of a Co site with a highly distorted environment coordinating two N atoms (2 ± 1) and one additional Co atom (1.0 ± 0.9) located at 2.54 ± 0.08 Å from the absorbing atom. The large uncertainties stem from a large number of fitting parameters that need to be considered to describe the environment of the Co site in this case. However, the obtained results confirm the presence of Co—Co bonds that could be associated with the presence of Co moieties (*e.g.*, Co dimers) or ultrasmall metallic clusters.

From the concentration profiles in Fig. 3 ▸(*b*) it is evident that the second component appears very quickly after the onset of CO_2_RR (within 5 min). After that, its concentration reaches ∼40% and it remains almost constant. On the other hand, the transformation of the first component into the third one is more gradual. After ∼35 min, the third component constitutes the majority of the chemical species, with a concentration of ∼60%. The obtained results agree well with our previous work (Hursán *et al.*, 2024 ▸), where we demonstrated, based on EXAFS analysis, that after 1 h of CO_2_RR ∼50% of the cobalt sites in the Co–N–C catalysts seem to aggregate and form metallic-like species. However, due to the limited information amount in EXAFS data for the TM-N-C systems and the lack of sufficient time resolution, our prior work could not provide definitive answers about the kinetics of the catalyst evolution, and, crucially, also about the nature of the remaining cationic Co species, which we believe are the main active sites for CO_2_RR. By focusing here on the analysis of time-resolved XANES data, we address this issue. Indeed, as demonstrated in our prior work (Hursán *et al.*, 2024 ▸), the concentration of metallic species depends also on the applied potential, and its increased content at lower cathodic potentials appears to correlate with the enhanced contribution of the parasitic hydrogen evolution reaction that is competing with CO_2_RR. This allows us to suggest that these species are not active for CO_2_RR, but the main active sites for the CO_2_ to CO conversion are instead associated with the third pure species, which preserves their singly dispersed cationic state, but, nonetheless, has a different Co environment than the single sites in the as-prepared catalyst. To clarify the local structure of the Co in these working sites, a XANES fitting was performed using the indirect machine learning approach.

**Figure 5:**
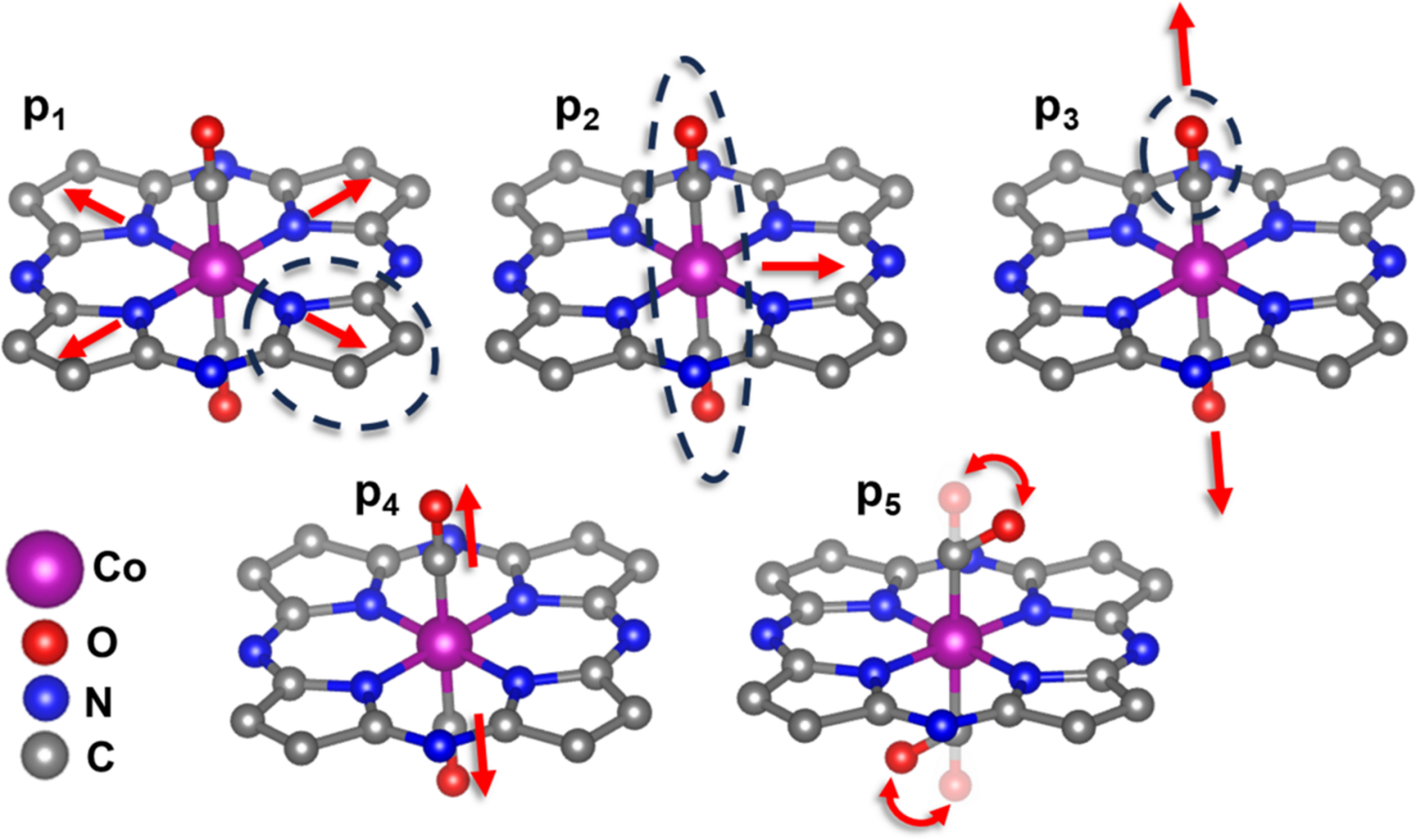
Set of structural deformations employed for the XANES fit of the third pure XANES component shown in Fig. 3 ▸(*a*).

The initial structure model was constructed based on the structure of the Co phthalocyanine complex (Crystallography Open Database, 2024 ▸) with the addition of two axial CO ligands with initial Co—CO distance of 2 Å and initial C—O distance of 1.1 Å (National Institute of Standards and Technology Database, 2024 ▸). To construct the training dataset, we distorted this initial structural model as shown in Fig. 5 ▸ and described in Table 1 ▸. We emphasize here that, since XAS is a local technique, we are not sensitive to the arrangement of atoms that are further away from the central Co atom (*e.g.*, the exact arrangement of C atoms in the carbon rings). The employed distortions are matching those used by us for the Ni–N–C case (Martini *et al.*, 2023 ▸). In total, ∼5000 distorted structures were generated and used to simulate the *ab initio* XANES spectra. Afterwards, these spectra were normalized by a common factor α = 0.00248, and used as the training set for the RBF regressor. The trained ML model has a prediction accuracy value higher than 0.94, indicating that the model yields the XANES spectra closely matching those obtained using the explicit *FDMNES* simulations.

Using the trained ML model, the XANES fit was performed in the energy range spanning from 7719 to 7795 eV, with the exclusion of the pre-edge region. The latter was necessary due to the lower accuracy of the *FDMNES* code in the simulations of this part of the XANES spectrum (Joly, 2021 ▸; Guda *et al.*, 2019 ▸).

**Figure 6:**
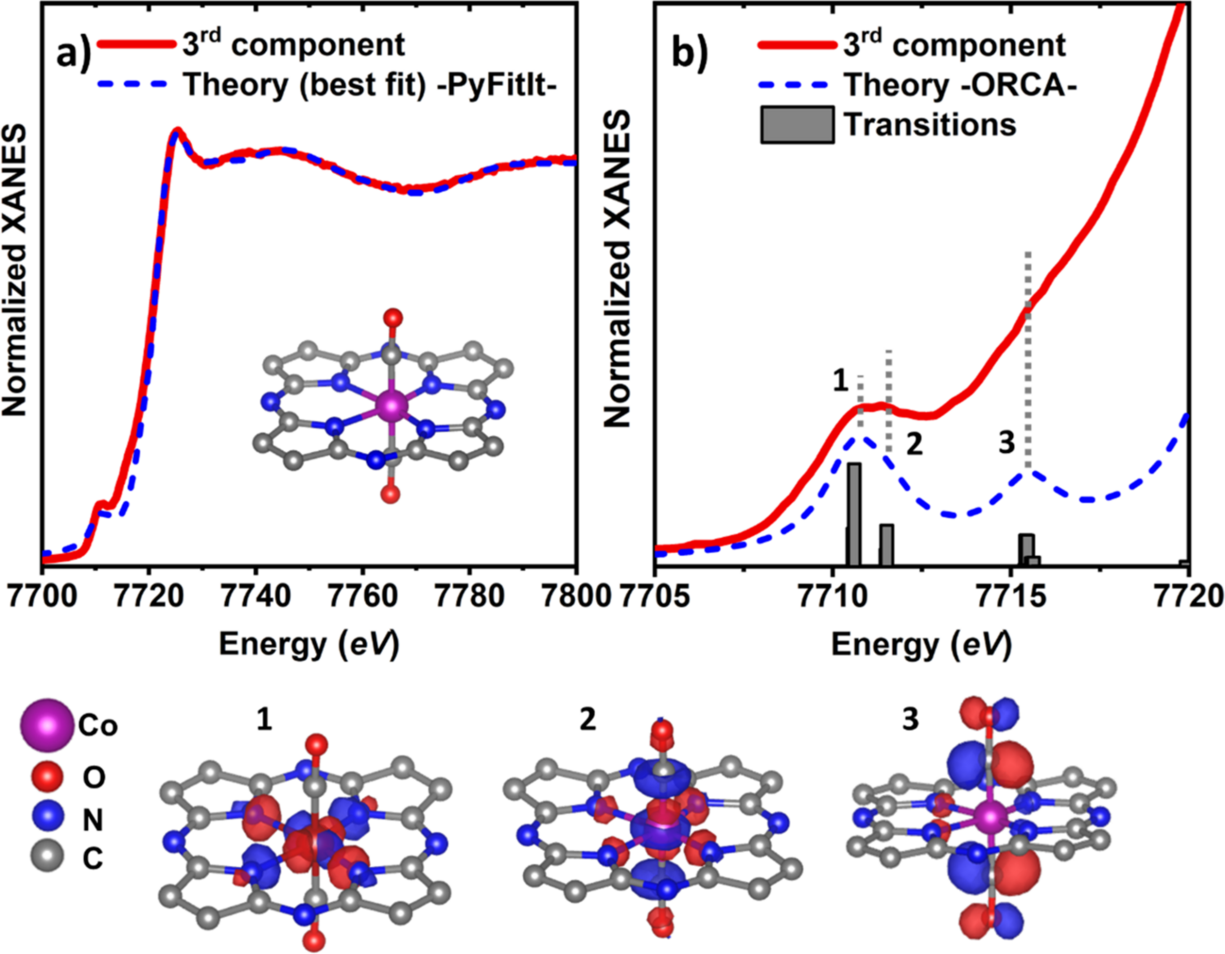
(*a*) Best-fit of the XANES third component showed in Fig. 3 ▸(*a*) using the machine learning indirect approach. (*b*) Reproduction of the pre-edge region of the third component using TDDFT calculations (quartet state). The grey bars represent the transition strengths involved in the total spectrum, while the numbers 1–3 indicate the principal (acceptor) natural transition orbitals (Martin, 2003 ▸). The calculated spectrum and the single transitions are shifted by 17.1 eV in order to have the best match with the experimental features of the pre-edge. For the three main orbital representations the iso-value is set to 0.06.

The XANES fitting results, together with the optimized structure, are reported in Fig. 6 ▸(*a*). The full list of the refined structural parameters can be found in Table S3. Table 2 ▸ reports the derived interatomic distances in the first coordination shell around Co, as well as the C—O interatomic distances and the ∠Co—C—O bonding angle.

One can see that the agreement between the XANES data extracted from the experimental dataset and the fitting results is very good, with an *R*_factor_ value of lower than 1%. The obtained interatomic distances, in turn, are comparable with the ones obtained for the Ni–N–C case (Martini *et al.*, 2023 ▸). The XANES fit shows that the Co atom is slightly displaced from the centre of the N_4_ square, resulting in two shorter and two longer Co—N_4_ bonds (with distances of 1.77 ± 0.03 Å and 2.12 ± 0.03 Å, respectively). Such symmetry breaking can be an indication that these singly dispersed Co sites are not distributed uniformly in the carbon support but are localized in the vicinity of some defects, as has been shown for similar systems elsewhere (see Fan *et al.*, 2020 ▸; Kramm *et al.*, 2012 ▸; Mou *et al.*, 2019 ▸; Wang *et al.*, 2018 ▸; Zheng *et al.*, 2018 ▸; Yan *et al.*, 2018 ▸; Rong *et al.*, 2020 ▸; Zhao *et al.*, 2017 ▸; Zhou *et al.*, 2023 ▸). Interestingly, while the average Co—N bond length in Co–N–C is slightly lower than the Ni—N bond length in the Ni–N–C (where the obtained interatomic distances for the shorter and longer Ni—N bonds were 1.82 and 2.17 Å, respectively), the difference between the longer and shorter bonds in both cases is ∼0.35 Å, suggesting a similar degree of the off-centre displacement. The axial CO bonds, in the case of Co–C–N, form a ∠Co—C—O angle of 178 ± 6°, with a Co—C distance of 1.71 ± 0.02 Å and a C—O distance of 1.25 ± 0.02 Å. Within the uncertainty, the C—O distance is the same for Co–N–C obtained for Ni–N–C, while the Co—C distance is slightly shorter than the Ni—C distance in Ni–N–C (1.78 Å), which can be attributed to the smaller size of the Co cation. As we mentioned in Section 2.3.2, the pre-edge region was not included in the fit. Nonetheless, its overall shape is reasonably reproduced by our fitting results, including the relative positions and the shape of the pre-edge peaks, see Fig. 6 ▸(*a*) and Fig. S8. This is particularly important, considering that this XANES part can be rich in spectroscopic details about the molecular structure of the Co site because it mainly involves the transitions of the Co 1*s* electrons to empty 3*d* states and to the unoccupied molecular orbitals of the possible adsorbates (Gallo *et al.*, 2011 ▸).

To exploit this sensitivity of the pre-edge, and to validate the obtained structural model, including the presence of CO adsorbates, the simulations of the pre-edge were performed using a TDDFT approach. The *ORCA* 5.0.4 code was employed and the refined Co–N–C structure model, obtained in *PyFitIt*/*FDMNES* simulations, was used as the input for the calculation. All the C terminal atoms of the carbon rings were saturated with H to ensure charge neutrality of the model. Both doublet (low spin) and quadruplet (high spin) spin states were considered. However, the differences between these obtained results for these two cases were found minimal, see Fig. S12, thus they could not be reliably discriminated within the energy resolution of our experiment.

Alternative spectroscopic techniques could be employed here, such as *K*_α_X-ray emission spectroscopy (Saveleva *et al.*, 2021 ▸; Saveleva, Kumar *et al.*, 2023 ▸). Overall, the quadruplet simulated spectrum shows a slightly better agreement with the experiment, and is thus reported in Fig. 6 ▸(*b*) together with the schematics of the acceptor orbitals responsible for the most intense spectroscopic transitions. We conclude that the first pre-edge peak at ∼7711 eV stems from two main transitions involving the Co core 1*s* orbital and two molecular orbitals (points 1 and 2 in Fig. 6 ▸) dominated mainly by the Co *e*_*g*_ orbitals characters: 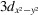 and 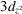, respectively. At the same time, the peak at ∼7716 eV is attributed to a metal-to-ligand charge transfer. The simulation shows, in fact, that it derives from the non-occupied states of the 2*p*(C)–2*p*(O) π* anti-bonding molecular orbital (point 3), thus confirming the presence of CO adsorbates.

## 4. Conclusions

In this work we demonstrate how the *operando* time-resolved XAS in combination with advanced data analysis approaches can be employed to probe the heterogeneous, dynamic structure of the TM-N-C catalysts. The combination of un­supervised ML approaches as PCA and TM methods allowed us to identify the main species available and co-existing in the working Co–N–C catalysts for the electrocatalytic CO_2_ reduction, and for the first time to track their kinetics. XANES data fitting, facilitated by supervised ML and XANES simulations within the TDDFT approach, in turn clarified the structure of the main active species for CO_2_ conversion to CO. We observed that the speciation of Co–N–C catalysts is more complex than that for the Ni–N–C catalyst, and, in particular, that the formation of reduced Co clusters plays an important role. The amount of reduced Co clusters, and the sizes of the metallic Co particles are likely to depend strongly on the details of the electrochemical system, including the sample preparation, metal loading, applied potential and duration of the experiment. The presence of these species, which we believe are not active for CO_2_RR but facilitate hydrogen evolution reaction, could explain the large discrepancies between the CO_2_RR activities for Co–N–C materials reported in the literature. Indeed, our analysis of the local structure of originally singly dispersed cationic Co species that coexist with multi-Co moieties under CO_2_RR conditions suggest that their structure is highly similar to that of active Ni–N–C catalysts. In particular, the interaction between Co and CO adsorbates can be directly observed in our XANES data. The obtained results thus show promise that Co–N–C catalysts can potentially reach similar CO_2_RR activities as their Ni-based counterparts, provided that the highly dispersed Co–N–C species can be maximized during CO_2_RR, for instance by their careful initial placement at/close defects on the N-doped C support matrix.

Intriguingly, our results suggest that the kinetics of the formation of metallic clusters and active single Co sites under CO_2_RR conditions are vastly different. The provided guidance from *operando* measurements with increased temporal resolution, involving sub-second quick-XAS measurements (Timoshenko *et al.*, 2022 ▸), thus would be highly instrumental for the rational design of these future experiments. On the other hand, the measurements of high-energy-resolution fluorescence-detected XANES (Saveleva, Retegan *et al.*, 2023 ▸), more sensitive to the geometry of the site because of the lower order of the core–hole lifetime broadening, would help to provide further details about the interactions between the metal site and adsorbates, yielding new insight into the working mechanism of TM-N-C catalysts.

## Supplementary Material

Additional details on the XANES and EXAFS analysis reported in the main text. DOI: 10.1107/S1600577524004739/ok5115sup1.pdf

## Figures and Tables

**Table 1:** List of structural parameters for the model shown in Fig. 5 ▸, employed in the fit of the third XANES component shown in Fig. 3 ▸(*a*)

Model used to describe the third Co *K*-edge XANES component		
*p*_1_	Contraction/expansion of the N_4_ square	[−0.2: +0.2] Å
*p*_2_	Shift of the Co atom and of the CO groups towards the edge of the N_4_ square	[0: +0.3] Å
*p*_3_	Contraction/expansion of the axial Co—C bonds	[−0.1: +0.2] Å
*p*_4_	Contraction/expansion of the C—O bonds.	[−0.2: +0.2] Å
*p*_5_	Co—C—O bond angle.	[135: 180]°

**Table 2:** Interatomic distances and Co—CO angle, obtained from the XANES fit The uncertainties are derived from the ones reported in Table S3 and are indicated in parenthesis.

Distance (average)/angle	Co *K*-edge XANES best-fit values
Misfit (*R*_factor_): 0.96%	
Co—C (C of the CO groups)	1.71 (2) Å
Co—N (two N atoms that are closer to Co)	1.77 (3) Å
Co—N (two N atoms that are further away from Co)	2.12 (3) Å
C—O (C and O of the CO groups)	1.25 (2) Å
∠Co—C—O bond angle	178 (6)°
