# Supplementary material for: Revealing the structure of the active sites for the electrocatalytic CO2 reduction to CO over Co single atom catalysts using operando XANES and machine learning
Source: J Synchrotron Radiat. 2024 Jun 25;31(Pt 4):741–50. doi: 10.1107/S1600577524004739 (PMC11226159; doi:10.1107/S1600577524004739)
Supplement: Supplementary file 1 [file s-31-00741-sup1.pdf]

JOURNAL OF
SYNCHROTRON
RADIATION

Volume 31 (2024)

Supporting information for article:

Revealing the structure of the active sites for the electrocatalytic CO2 reduction to CO over Co single atom catalysts using *operando* XANES and machine learning

Andrea Martini, Janis Timoshenko, Martina Rüscher, Dorottya Hursán, Mariana C. O. Monteiro, Eric Liberra and Beatriz Roldan Cuenya

S1. Comparison of the *operando* XANES spectra with references

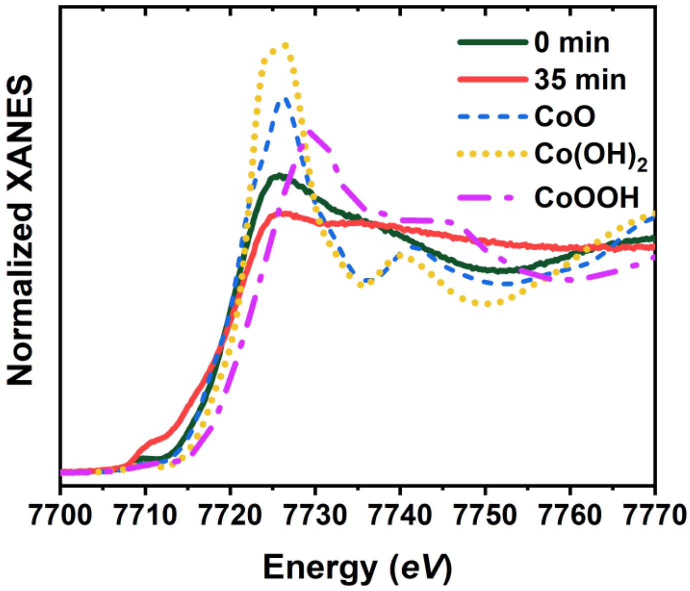

Figure S1 Operando Co K-edge XANES spectra for Co-N-C catalyst at the beginning and at the end of the CO_2RR reaction (performed at -1.2 V_{RHE} in 0.1 M $KHCO_3$ electrolyte) and the reference XANES spectra for CoO (Co oxidation state +2), $Co(OH)_2$ (Co +2) and finally CoOOH (Co +3).

S2. XANES pre-edge and white line region of the three *pure* XANES

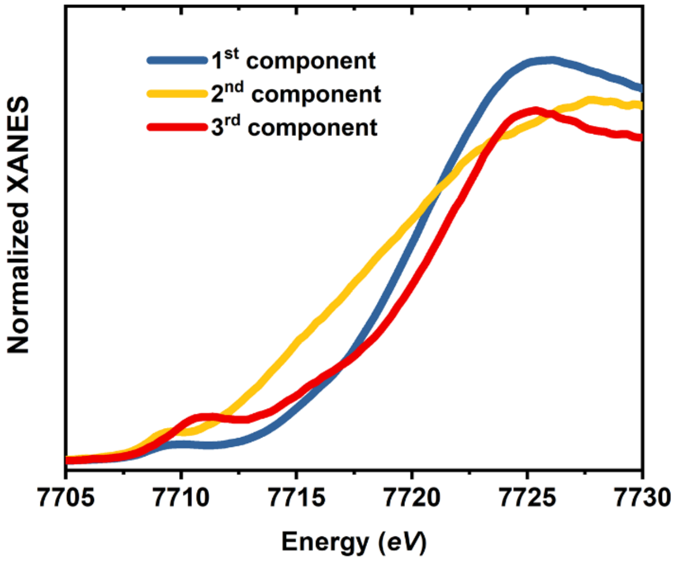

Figure S2 Pre-edge and white-line region of the three XANES spectra belonging to the pure species extracted using the TM method. The full range XANES data are reported in **Figure 3** of the main text.

S3. FDMNES convolution parameters

The parameters reported in Table S1 were selected for the convolution of the calculated FDMNES spectra employing an energy dependent arc-tangent shape of the Lorentzian profile and optimised over the Co XANES K-edge of the CoO reference spectrum.

Table S1 FDMNES convolution parameters chosen for the theoretical Co K-edge XANES spectra.

Gamma hole (eV)	Ecent (eV)	Elarge (eV)	Gamma max (eV)	E Fermi (eV)	Energy shift (eV)
2.70	28.7	16.8	19.4	7709.5	142

S4. Extracted EXAFS signals for the first and third component

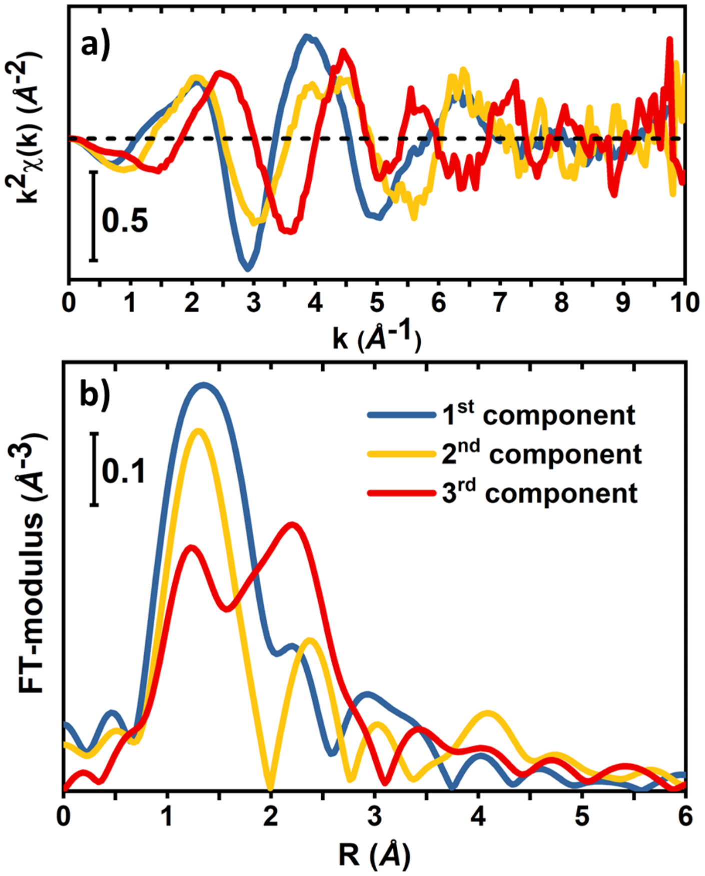

Figure S3 (a) EXAFS signals weighted by a k^2 factor, extracted for the three pure species. (b) Magnitude of their Fourier transformed signals. The Fourier transforms are not corrected for the phase shift.

S5. Wavelet Transform and EXAFS fit of the 2nd component

S5.1. Wavelet Transform

The WT was carried out using the following equation: $W_{\psi}(k,a) = \frac{1}{\sqrt{a}}\int_{-\infty}^{+\infty} k'^{2}\chi(k')\psi^{*}\left(\frac{k'-k}{a}\right)dk'$ (Timoshenko & Kuzmin, 2009; Funke *et al.*, 2005). Here ψ is the so-called mother wavelet function. One of the most suitable mother wavelet functions for the EXAFS analysis is the Morlet wavelet: $\psi(k) = \frac{1}{\sqrt{2\pi s}}\exp(i\eta k)\exp\left[-\frac{k^2}{2s^2}\right]$. In this work, the Morlet parameters with $s = 1$ (value for the width of the Gaussian envelope), and $\eta = 6$ (frequency of the harmonic function) were chosen, allowing the optimal resolution (Funke *et al.*, 2007) in both k- and R- spaces for the second coordination shell features. For the Morlet wavelet transform, the scale parameter a can be linked to the signal frequencies R values as $a = \eta/2R$.

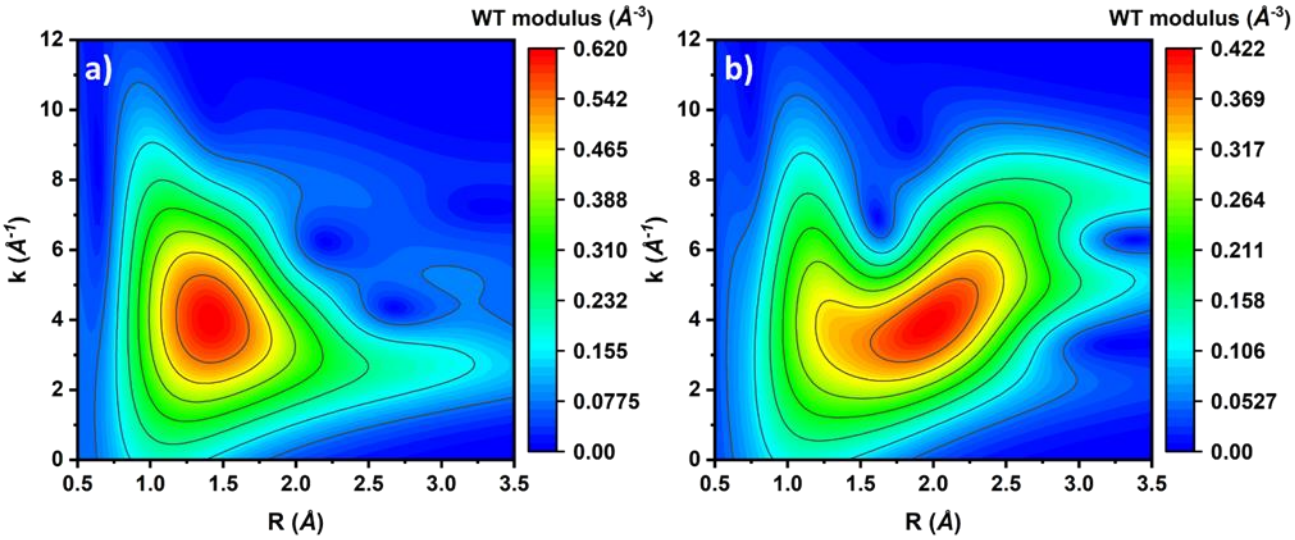

Figure S4 Moduli of the Morlet wavelet transform calculated for the EXAFS signal of the 1st and 3rd pure species. The wavelet resolution parameters σ and η were set to 1 and 6, respectively. Differently from the 2nd species, for both these two cases, the second shell wavelet lobes (2-2.7 Å) are located mainly in the range within 4 and 6 Å suggesting mainly the existence of light scatterers. The wavelet transforms are not corrected for the phase shift.

S5.2. EXAFS fit

The EXAFS fit of the extracted 2nd component was performed in the R-range between 1 and 3.5 Å. The k^2-weighted signal was Fourier Transformed using a Hanning window defined in the k-space within 2.3 and 9 $Å^{-1}$. The fit was realised in Artemis (Ravel & Newville, 2005) considering 3 SS paths: Co-N, Co-C and Co-Co. The photoelectron scattering phases and amplitudes for the first two were calculated by the FEFF 6.0l code (Ankudinov *et al.*, 1998) for the Co phthalocyanine complex (Crystallography-Open-Database) while for the third paths, calculations for the metallic Co were performed. S_0^2 factor was fixed to 1 for all the paths,

on the basis of the EXAFS fit performed for the CoO reference (Hursán *et al.*, 2023). In order to reduce the correlation between the coordination numbers and the Debye-Waller (DW) factors, the DW for the Co-Co path, was set to 0.008 Å^2, a value already used for dispersed Cu sites in zeolites frameworks (Martini, Signorile, *et al.*, 2020; Deplano *et al.*, 2021; Martini *et al.*, 2017). At the same time, it has been assumed that the N and C have the same DWs. The following variables were then guessed and refined through the EXAFS fit: the coordination numbers of the N, C and Co atoms (N_N, N_C and N_{Co}), their corresponding distances from the Co absorber, the DW of the N/C atoms and finally a common reference energy shift parameter ΔE_0. The best-fit results are reported in **Table S2** while the comparison between the EXAFS 2nd component and the related best-fit is shown in **Figure S5.**

Table S2 EXAFS best fit parameters obtained from the analysis of the EXAFS spectrum of the 2nd component. Uncertainties of the last digit are given in parentheses.

Fitting parameters	Best fit results
$\%R_{factor}$: 1.4	
ΔE_0 (eV)	-3(6)
N_N	2(1)
N_C	1.0(9)
N_{Co}	2(2)
$\sigma_{N/C}$	0.005(7)
R_{Co-N} (Å)	1.91(5)
R_{Co-C} (Å)	2.8(2)
R_{Co-C} (Å)	2.54(8)

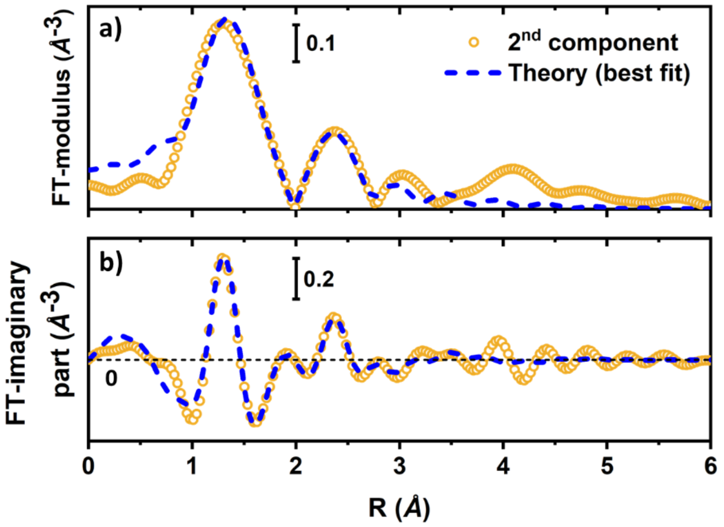

Figure S5 Comparison between the experimental pure EXAFS spectrum belonging to the 2nd component and its best fit: (a) magnitude, (b) imaginary part.

S6. XANES fit

Table S3 XANES best-fit structural parameters for the model shown in **Figs. 5** and **6**(a) of the main text.

Fitting parameters	XANES best-fit values
Misfit (R_{factor}): 0.96%	
p_1 (Å)	0.02(2)
p_2 (Å)	0.25(2)
p_3 (Å)	-0.04(2)
p_4 (Å)	0.15(2)
p_5 (°)	178(6)

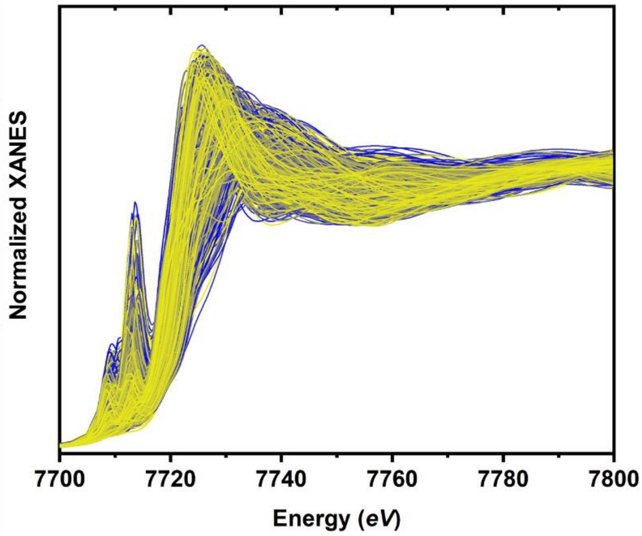

Figure S6 Representative XANES theoretical set composed by 1000 spectra (generated using the FDMNES code (Guda et al., 2015; Joly, 2001)) used to train the Radial Basis Function Regressor algorithm implemented in PyFitIt (Martini, Guda, et al., 2020) following the adaptive sampling approach (Tereshchenko et al., 2022) in the ranges of **Table 1**.

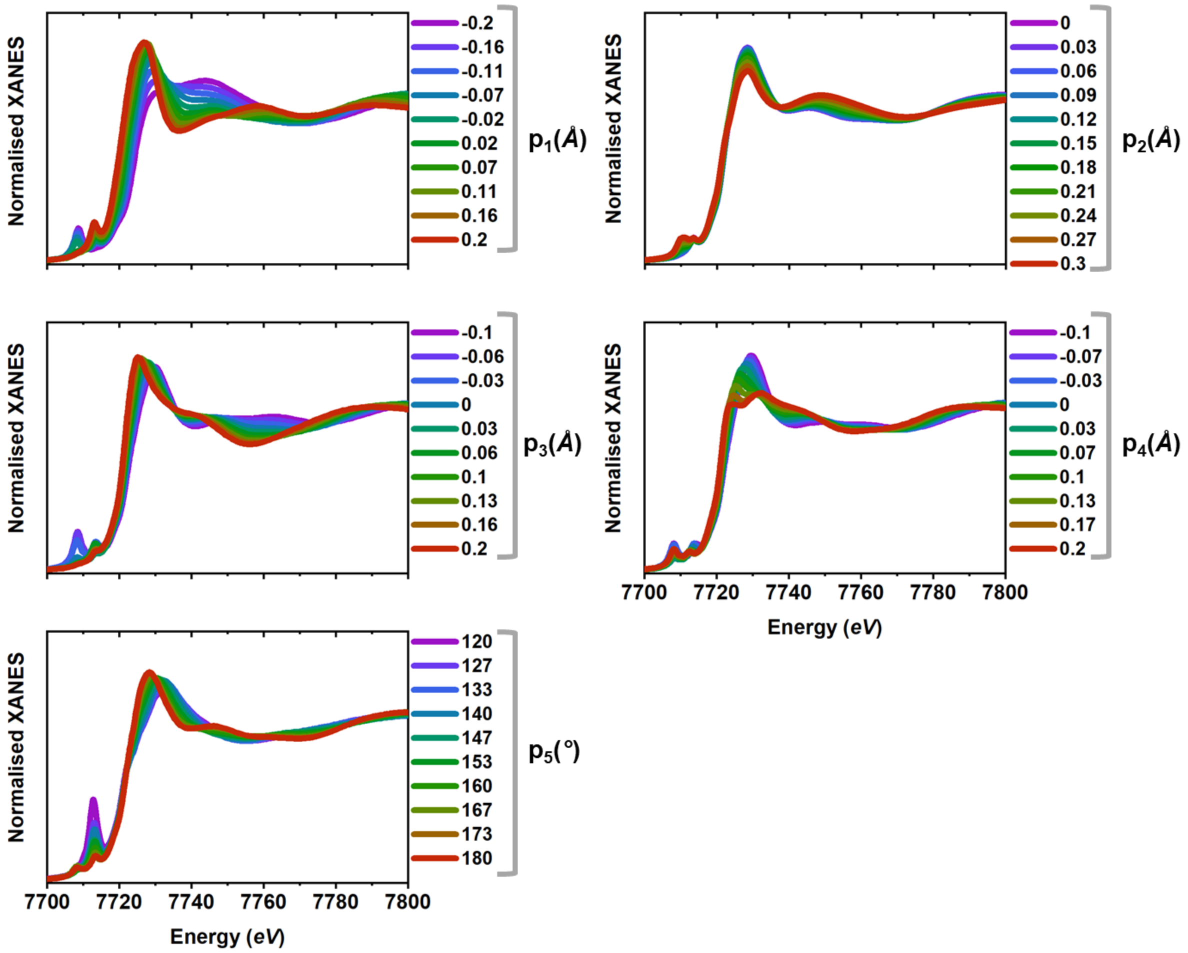

Figure S7 XANES changes associated to some (arbitrary) selected variation of each structural parameter shown in **Figure 5** of the main text.

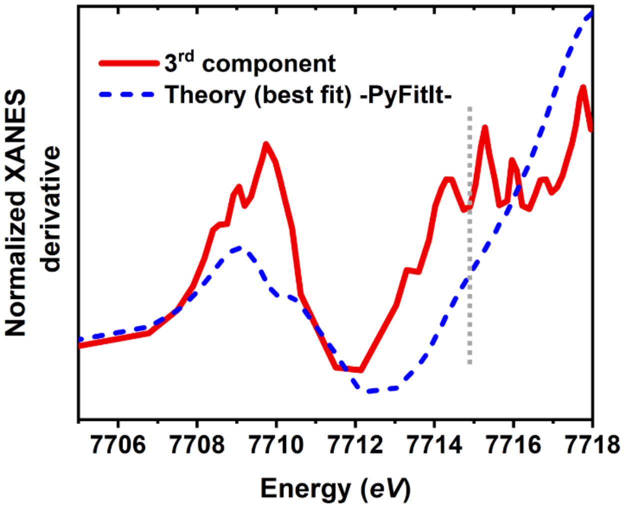

Figure S8 First derivative plot showing the pre-edge-region of **Figure 3**(a) of the main text together with the one reproduced using the PyFitIt code (i.e. FDMNES), see **Figure 6**(a). The dotted grey line indicates here the presence of a flex point deriving from a weak experimental and theoretical XANES feature at ca. 7714.5 eV.

S6.1. XANES fit of the first component

Figure S9 shows the Co structure employed in the fit of the 1st *pure* spectrum, corresponding to the as-prepared catalyst (**Figure 3** of the main text). To fit the Co K-edge XANES, we used the analogous approach to that discussed in the main text for the 3rd *pure* species. For the as-prepared state of the catalyst, the training sets contained ca. 1000 theoretically generated XANES, which allowed us to achieve an accuracy higher than 0.96, indicating a very good level of approximation. The comparison between the experiment and the best-fit is shown in Figure S10, while the best-fit parameters are reported in Table S5 and Table S6.

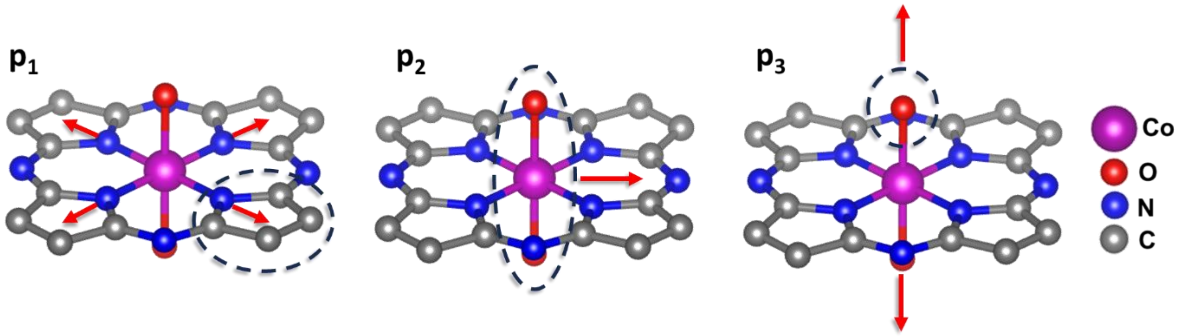

Figure S9 Set of structural deformations employed for the XANES fit of the 1st pure XANES component shown in **Figure 3**(a) and described in Table S4.

Table S4 List of structural parameters for the model shown in Figure S9. These were employed in the fit of XANES spectrum for the 1st pure species (as-prepared state of the catalyst).

**Model used to describe the 1st Co K-edge XANES component**		
p_1	Contraction/expansion of the N_4 square.	[-0.2 : +0.2] Å
p_2	Shift of the Co atom and of the CO groups towards the edge of the N_4 square.	[0 : +0.3] Å
p_3	Contraction/expansion of the axial Co-O bonds.	[-0.1 : +0.2] Å

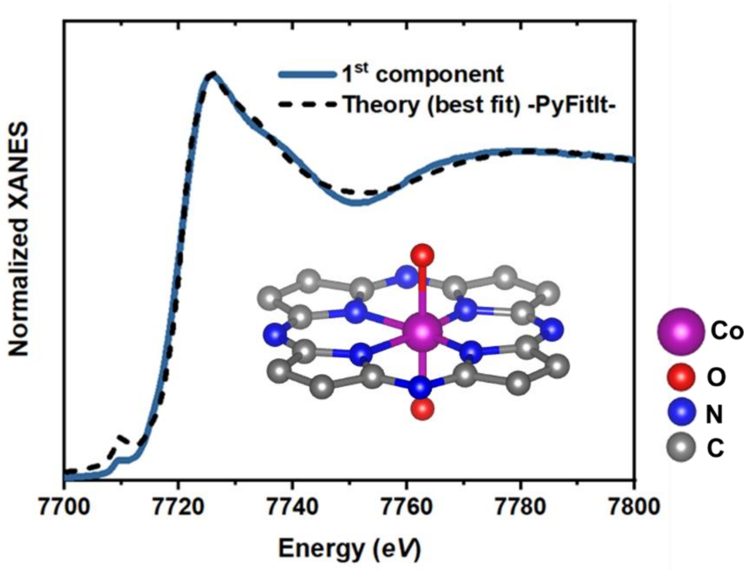

Figure S10 (a) Best-fit of the XANES spectrum for the 1st pure species (as-prepared state of Co-N-C catalyst) obtained using the *indirect* machine learning approach with a normalization parameter α of 0.025. The inset shows the final structure obtained in the XANES fit.

Table S5 XANES best-fit structural parameters for the model shown in Figure S9 and Figure S10.

**Fitting parameters**	**XANES best-fit values**
Misfit (R_{factor}): 1.7 %	
p_1 (Å)	-0.03(3)
p_2 (Å)	0.22(3)
p_3 (Å)	0.02(3)

Table S6 Interatomic distances obtained from the XANES fit. The uncertainties are derived from the ones reported in Table S5 and are indicated in parenthesis.

**Distances (average)/Angle**	**Co K-edge XANES best-fit values**
Misfit (R_{factor}): 1.7 %	
Co-O	2.02(3)
Co-N (two N atoms that are closer to Co)	1.74(4)
Co-N (two N atoms that are further away from Co)	2.05(4)

S7. Reverse Monte Carlo EXAFS fit of the first and third component

To check whether the structure models derived based on XANES data fitting for the 1st and 3rd components (showed in **Figure 6** and in Figure S10) are consistent also with the available EXAFS data, we performed reverse Monte Carlo (RMC) simulations as implemented in the EvAX code (Timoshenko *et al.*, 2014, 2012). In the RMC-EXAFS approach, we start with a 3D structure model obtained using the XANES fitting procedure and slightly move the atoms in the model around their initial positions in a random process in order to include the thermal and static disorder effects. The maximal allowed atom displacements from the starting atomic positions in the RMC simulations were set to 0.4 Å; thus, the overall 3D structure of the material and coordination numbers do not change in the RMC-EXAFS fit. The RMC approach allows us to fit EXAFS data and accounts explicitly for the contributions of distant coordination shells and multiple scattering effects.

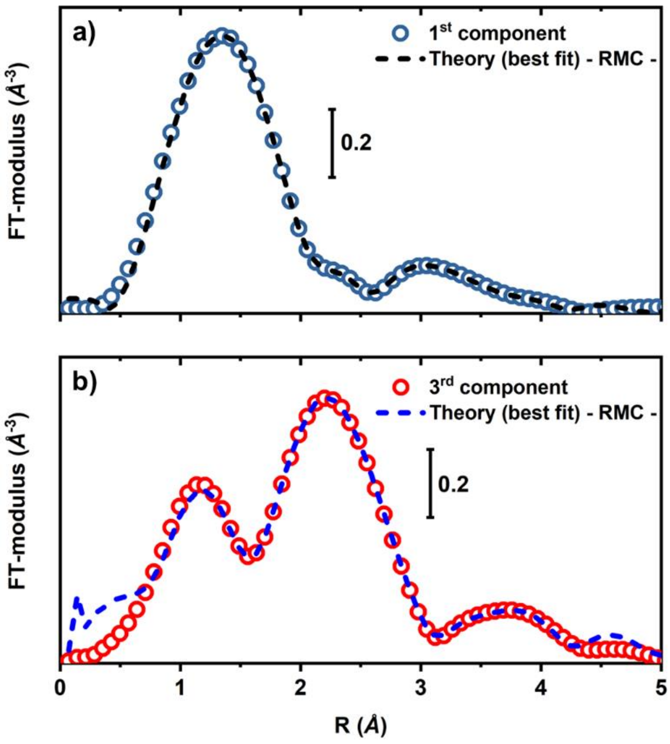

Figure S11 (a) Results of the RMC-EXAFS simulations using the EvAX code (Timoshenko et al., 2014, 2012). Comparison of Fourier-transformed experimental Co K-edge EXAFS spectra for the 1st and 3rd component with the corresponding RMC-EXAFS results for the final structure models obtained through the XANES fits. The Fourier transforms are carried out in the k-range between 2 and 9 $Å^{-1}$. RMC fits are carried out in k- and R-spaces simultaneously using the wavelet transform, in the k-range between 2 and 9 $Å^{-1}$ and in the R-range between 0.8 and 4.5 Å, including multiple scattering contributions with up to 5 Å.

Figures S11(a, b) shows the RMC fits for the 1st and 3rd component. RMC simulations yield structure models that are in an excellent agreement with the experimental EXAFS data, with R_{factor} values of 0.01% (see **Figure S11**(a)) and 0.16% (see **Figure S11**(b)), respectively. Thus, this confirms that the structure models obtained from the XANES fitting are in a full agreement with the available experimental EXAFS data.

S8. XANES pre-edge *ab initio* simulation of the 3rd component (doublet case)

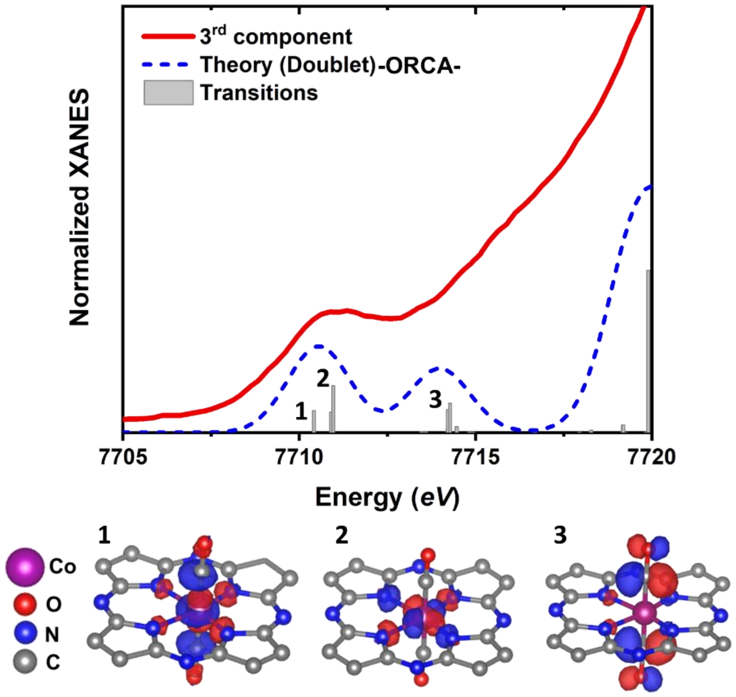

Figure S12 Reproduction of the pre-edge region of the 3rd component using TDDFT calculations (doublet state). The grey bars represent the transition strengths involved in the total spectrum, while the numbers 1-3 indicate the principal (acceptor) natural transition orbitals (Martin, 2003). The calculated spectrum and the single transitions are shifted by 17.1 eV as for the quartet state showed in **Figure 6**(b). For the three main orbital representations the iso-value is set to 0.06.

From Figure S12, transitions (**1**) and (**2**) are dominated by the e_g orbitals characters, here $3d_{z^2}$ $3d_{x^2-y^2}$ while (**3**) shows the electron charge transfer from the Co 1s to a molecular orbital strongly affected by the 2p(C)-2p(O) π^*character.

References

Ankudinov, A. L., Ravel, B., Rehr, J. J. & Conradson, S. D. (1998). *Phys. Rev. B* **58**, 7565-7576.

Crystallography-Open-Database (2024). *http://www.crystallography.net/cod/2100746.html*

Deplano, G., Martini, A., Signorile, M., Borfecchia, E., Crocellà, V., Svelle, S. & Bordiga, S. (2021). *Angewandte Chemie-International Edition* **60**, 25891-25896.

Funke, H., Chukalina, M. & Scheinost, A. C. (2007). *Journal of Synchrotron Radiation* **14**, 426-432.

Funke, H., Scheinost, A. C. & Chukalina, M. (2005). *Phys. Rev. B* **71**.

Guda, S. A., Guda, A. A., Soldatov, M. A., Lomachenko, K. A., Bugaev, A. L., Lamberti, C., Gawelda, W., Bressler, C., Smolentsev, G., Soldatov, A. V. & Joly, Y. (2015). *Journal of Chemical Theory and Computation* **11**, 4512-4521.

Hursán, D., Timoshenko, J., Ortega, E., Jeon, H. S., Rüscher, M., Herzog, A., Rettenmaier, C., Chee, S. W., Martini, A., Koshy, D. & Roldán Cuenya, B. (2023). *Advanced Materials* **n/a**, 2307809.

Joly, Y. (2001). *Phys. Rev. B* **63**, 125120.

Martin, R. L. (2003). *J. Chem. Phys.* **118**, 4775-4777.

Martini, A., Borfecchia, E., Lomachenko, K. A., Pankin, I. A., Negri, C., Berlier, G., Beato, P., Falsig, H., Bordiga, S. & Lamberti, C. (2017). *Chemical Science* **8**, 6836-6851.

Martini, A., Guda, S. A., Guda, A. A., Smolentsev, G., Algasov, A., Usoltsev, O., Soldatov, M. A., Bugaev, A., Rusalev, Y., Lamberti, C. & Soldatov, A. V. (2020). *Computer Physics Communications* **250**, 1-15.

Martini, A., Signorile, M., Negri, C., Kvande, K., Lomachenko, K. A., Svelle, S., Beato, P., Berlier, G., Borfecchia, E. & Bordiga, S. (2020). *Phys. Chem. Chem. Phys.* **22**, 18950-18963.

Ravel, B. & Newville, M. (2005). *Journal of Synchrotron Radiation* **12**, 537-541.

Tereshchenko, A., Pashkov, D., Guda, A., Guda, S., Rusalev, Y. & Soldatov, A. (2022). *Molecules* **27**, 1-13.

Timoshenko, J. & Kuzmin, A. (2009). *Computer Physics Communications* **180**, 920-925.

Timoshenko, J., Kuzmin, A. & Purans, J. (2012). *Computer Physics Communications* **183**, 1237-1245.

Timoshenko, J., Kuzmin, A. & Purans, J. (2014). *Journal of Physics-Condensed Matter* **26**, 1-15.
